# *Cryptococcus neoformans* in the sputum: a noninvasive diagnostic method

**DOI:** 10.1590/0037-8682-0250-2024

**Published:** 2025-01-27

**Authors:** Simone Rachid de Souza, Diogo Goulart Corrêa

**Affiliations:** 1 Universidade Federal do Rio de Janeiro, Departamento de Patologia, Rio de Janeiro, RJ, Brasil.; 2 Universidade do Estado do Rio de Janeiro, Departamento de Diagnóstico por Imagem, Rio de Janeiro, RJ, Brasil.; 3 Universidade Federal Fluminense, Departamento de Radiologia, Niterói, RJ, Brasil.

**FIGURE 1:**
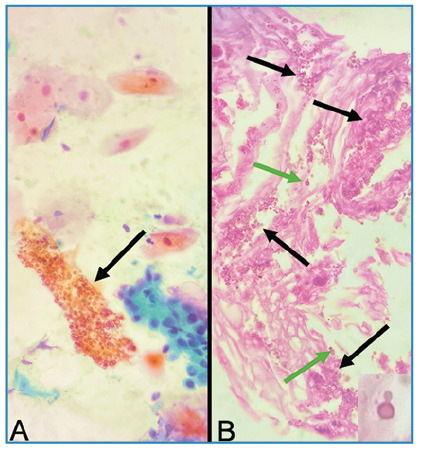
Cytopathological characteristics of sputum specimens. **(A)** Rounded fungal structures with thick capsules compatible with *Cryptococcus spp*. (arrow, 400× magnification, Papanicolaou staining). **(B)** Similar fungal structures (black arrows) showing narrow-based budding (green arrows and detail) in the cell block (400× magnification, hematoxylin and eosin staining).

**FIGURE 2:**
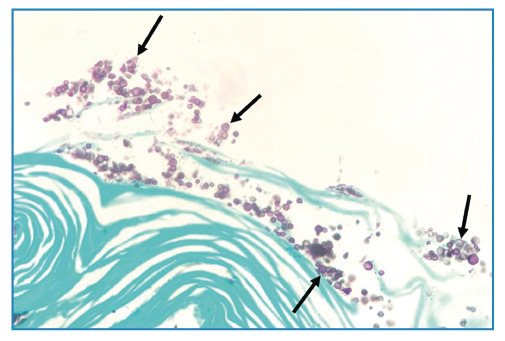
Grocott’s methenamine silver staining of the sputum cell block confirmed the presence of *Cryptococcus* yeasts (arrows; 400× magnification).

A 76-year-old HIV-positive man presented with a 2-month history of productive cough, dyspnea, and fever. Chest computed tomography revealed cavitation associated with small centrilobular nodules in the upper lobe of the left lung. Cytopathological analysis of induced sputum samples revealed columnar cells, numerous alveolar macrophages, and numerous rounded fungal structures of various sizes with thick capsules and sometimes budding compatible with *Cryptococcus spp*. (Figure 1). Grocott-Gomori methenamine silver staining was positive (Figure 2), and the sputum culture was positive for *Cryptococcus neoformans*.

*Cryptococcus neoformans* is present in soil and bird feces, and infects humans when inhaled, mainly affecting those with immunodeficiencies^1^. Cryptococcosis particularly affects the central nervous system and lungs of patients with acquired immunodeficiency syndrome. It may present as skin lesions, and disseminated forms of the disease exist^2^ ^,^ ^3^. Although the lungs are the primary sites of infection, their involvement is usually mild and asymptomatic. In immunosuppressed individuals, *Cryptococcus spp.* evoke virtually no inflammatory reactions; however, in non-immunosuppressed individuals and those with prolonged illness, the fungi induce a chronic granulomatous reaction^3^.

*Cryptococcus* appear as yeasts with diameters of 3-10 µm and highly characteristic thick gelatinous capsules with narrow-based budding^3^. Although induced sputum examination has shown only 57.5% sensitivity and 42.9% specificity for the diagnosis of lung infections in HIV-positive patients, its positive predictive value was reported to be 87.1%, indicating that it can help diagnose infectious diseases^4^.
